# Synthesis and characterization of nanoceria for electrochemical sensing applications

**DOI:** 10.1039/d1ra00637a

**Published:** 2021-04-30

**Authors:** Yeni Wahyuni Hartati, Seda Nur Topkaya, Shabarni Gaffar, Husein H. Bahti, Arif E. Cetin

**Affiliations:** Department of Chemistry, Faculty of Mathematics and Natural Sciences, Universitas Padjadjaran Indonesia yeni.w.hartati@unpad.ac.id; Department of Analytical Chemistry, Faculty of Pharmacy, Izmir Katip Celebi University Turkey; Izmir Biomedicine and Genome Center Izmir Turkey

## Abstract

Nanoceria (cerium oxide nanoparticles: CeO_2_-NPs) has received significant attention due to its biocompatibility, good conductivity, and the ability to transfer oxygen. Nanoceria has been widely used to develop electrochemical sensors and biosensors as it could increase response time, sensitivity, and stability of the sensor. In this review, we discussed synthesis methods, and the recent applications employing CeO_2_-NPs for electrochemical detection of various analytes reported in the most recent four years.

## Abstract 2

Nanoceria (cerium oxide nanoparticles: CeO_2_-NPs) has received significant attention due to its biocompatibility, good conductivity, and the ability to transfer oxygen.

## Graphical abstract

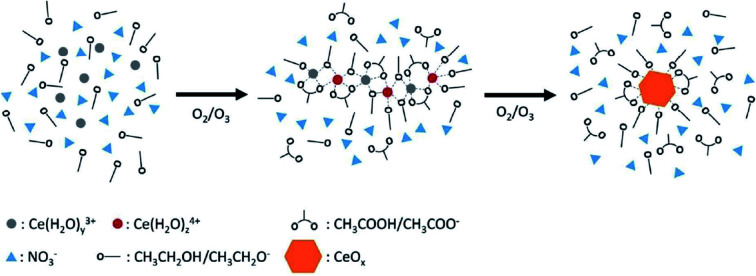

## 1 Introduction

Cerium is a member of the lanthanide metals, including bastnäsite, allanite, cerite, monazite, euxenite, and xenotime), and it is the most abundant of rare-earth metals found in Earth's crust.^1^ Oxide of cerium is called ceria, and has two oxidation states Ce^4+^ (CeO_2_) and Ce^3+^ (Ce_2_O_3_). CeO_2_ has a cubic fluorite structure, where each Ce^+^ cation is surrounded by 8 oxygen anions, and each anion has a Ce^+^ tetrahedral cation.^2^ Oxidation potentials and numbers of electronegative oxygen atoms are related to their ability to interact with bio-macromolecules, *e.g.*, proteins, nucleic acids, and others in specific medium.^3^

Cerium(iv) oxide is also known as cerium dioxide, ceria oxide, ceria dioxide and ceria, and has been widely used as an antibacterial agent, biosensor, catalyst, fluorescent material, and for agricultural applications.^4–7^ Nanoceria is a cerium oxide with a size between 1 and 100 nm, and has diverse applications such as sensors and drug delivery^8,9^ due to its attractive properties, *e.g.*, biocompatibility, chemical and thermal stability, high oxygen storage capacity, good superficial electrical diffusivity, and conductivity.^10–12^ Nanoceria can be also used for transducers thanks to its high sensitivity.^13–15^ The interaction between adsorbed molecules and the cerium oxide surface is dependent on crystal surface and plane properties of the nanoceria. For instance, spherical crystalline nanoceria electrodes show higher discharge capacity than carbonaceous electrode.^16^ Physicochemical properties of nanoparticles depend on the synthesis methods, *e.g.*, different methods have been used to produce nanoceria in different morphology such as size, shape, distribution, and agglomeration. In nanoceria synthesis, various precursors have been used, *e.g.*, raw materials such as cerium nitrate, cerium chloride, cerium sulphate which are then converted to cerium oxide.^17^

Physicochemical properties of nanoceria play an important role in the ultimate interaction with bioreceptors, target cells, and others chemical or biochemical species. Physicochemical and surface properties of nanoceria, *e.g.*, synthesis methods controlling their biological activity as an inactive, antioxidant, or pro-oxidant, have reviewed in detailed.^18^ A comparative evaluation on photocatalytic efficiencies with physicochemical properties of nanoceria,^19^ toxicity effect,^20^ adsorption of metals onto nanoceria, and biosensing applications with nanoceria have been also performed in various reviews in literature.

In this paper, synthesis and characterization methods, and applications of nanoceria for electrochemical bio-detection of various analytes reported in the last four years (2017–2020) were reviewed.

## 2 Synthesis of nanoceria

Synthesis of nanoceria can be carried out by various physical and chemical processes such as precipitation, hydrothermal, sol–gel, solvothermal, and green synthesis. Different methods can produce nanoceria with different forms, shapes, patterns, and sizes. Correlation of nanoceria between physical and chemical properties, as well as their sizes and surface morphologies is extremely important for controllable synthesis of nanocrystals with specific dimensions and shapes. Sensing capability of nanoceria is also strongly dependent on its shape and size.

Chemical and physical methods are effective, and have faster synthesis time than biological processes. However, these methods have some disadvantages, *e.g.*, the use of hazardous chemicals which can generate toxic waste, while the physical methods are energy intensively, expensive, and harmful to the environment. In addition, nanomaterials employed in these methods need to modified for biocompatibility. Green synthesis becomes a popular method for the synthesis of nanomaterials, including nanoceria. Recently, various natural extracts have been used as bio-reductant and chelating agent. Furthermore, plant extracts, algae, fungi and bacteria can be used for the biosynthesis of nanoceria. Nanoparticles produced through green synthesis exhibit enhanced biocompatibility compared to physically or chemically synthesized ones as they are naturally reduced and stabilized by plant phytochemicals.^21^ However, the use of extracts except plants have limitation regarding to biosafety concerns, and need sterile conditions and maintenance of microbial cultures. Therefore, plants are preferred for extraction by providing a simple platform for green synthesis method of nanoparticles.

### 2.1 Precipitation method

Precipitation is very favorable technique due to its simplicity for synthesizing nanoceria at room or high temperature. The process does not require washing and purification steps. Cerium nitrate hexahydrate is mostly used as precursors for nanoceria synthesis under alkaline conditions, *e.g.*, aqueous ammonia or sodium hydroxide were used.^22–24^ Process of nanocrystal formation is carried out at room temperature, while calcining at various temperatures.

Nanoceria could be synthesized from cerium nitrate hexahydrate with poly(ethylene)imine (PEI) as a complexing agent and sodium salt of carboxy-methylated poly(ethylene)imine (Trilon P) as a dispersing agent.^25^ A precipitation method was developed with the use of cerium nitrate hexahydrate with poly vinyl pyrrolidone (PVP), poly vinyl alcohol (PVA), and ammonia as precursors.^26^

Cerium(iii) chloride heptahydrate with ammonium hydroxide can form ceria nanorods, through a stirring procedure.^27,28^ Cerium sulphate was used as a precursor in basic conditions, *e.g.*, ammonia water^29^ and sodium hydroxide.^30^ Matin *et al.* performed calcination at two different temperatures, where the shape, size, and distribution of nanoceria strongly influence the surface properties.^29^ Ultrasound assisted-precipitation in the synthesis of ceria nanoparticles could be achieved *via* the reduction of cerium nitrate hexahydrate at room temperature. Different solvents have been used, *e.g.*, methanol, ethylene glycol, water, and isopropyl alcohol, without the addition of a capping agent. Cerium nitrate hexahydrate was dissolved in distilled water and sodium hydroxide was added drop wise under sonication. The mixture was then irradiated with sonication, and nanoceria could be synthesized in the size of 4–8.^31^

### 2.2 Hydrothermal method

Hydrothermal method is the most preferred method for the synthesis of metals, metal oxides, and metal composites with variety of crystalline morphologies. It involves hydrolysis of metal salt using water as solvent, and condensation of metal hydroxide to produce ultrafine metal or metal oxide particles.^24^ Chemical reactions usually occur in under pressure. Particles in desired size and shape can be produced in hydrothermal process with the control of parameters, *e.g.*, pH, reaction time, temperature, type of solvent and solute concentration. Nanoceria could be synthesized with hydrothermal method using various precursors of cerium salts with particular modifications, *i.e.*, different morphologies of nanoceria have been developed. For instance, ceria nanorods were synthesized from cerium hexahydrate and urea with surfactant-free hydrothermal approach.^32^ Single crystalline mesoporous cerium oxide nanospheres were produced with CeCO_3_OH by controlling the amount of urea as a structural directing agent.^33^ Shape-specific nanoceria synthesized *via* hydrothermal method with cerium nitrate as precursors were precisely controlled by molarity of sodium hydroxide, reaction temperature, and pH.^34–37^ Addition of surfactants as a stabilizer agent can also control the overall shape of nanomaterials, and have critical roles on surface adsorption of surface active molecules. An 8–20 nm ceria nanocubes have been synthesized with cerium nitrate (precursor), and ammonia with TritonX-100 (surfactant).^38^ In another approach, adjusting pH of CeCl_3_ solution was realized by ammonia, *e.g.*, a white precipitate was formed to produce 40 nm nanoceria.^39^ An ultrasonic-assisted hydrothermal method was applied to produce ceria nanorods using cerium. At low pH, samples consist of nanoparticles, while at high pH, rod length increased, *e.g.*, cerium oxide nanorods were formed. Furthermore, Ce(OH)_3_ nuclei were formed right after Ce^3+^ ions were mixed with NaOH solution.^40^

### 2.3 Combustion method

Solution combustion synthesis combines combustion synthesis and reactive solution. Typically, a reaction of an oxidizer (usually metal nitrates) and a fuel cause releasing of large amounts of gases and heat. Fuels can be classified based on their type of reactive groups, *e.g.*, hydroxyl, amino, and carboxyl. Fuel-to-oxidizer ratio is one important parameter for determining stoichiometry and the morphology of the nanoparticles. Cerium nitrate hexahydrate (oxidizer) and nanoceria (precursor) reacted with glycine (fuel) under various conditions in ammonium nitrate that produced 6–30 nm nanoceria.^41,42^ The reaction of ceria formation from cerium nitrate with ammonium nitrate and glycine under combustion reaction could be written as Ce(NO_3_)_3_·6H_2_O_(aq)_ + 2C_2_H_5_O_2_N_(aq)_ + NH_4_NO_3(aq)_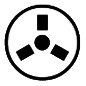 CeO_2(s)_ + 4CO_2(g)_ + 13H_2_O + 7/2N_2(g)_. The aqueous solution containing redox mixture in glassware then was heated to 150 °C, *e.g.*, after boiling, foams undergoes combustion with a flame to produce ceria.^43^ Solution combustion method was used for synthetizing nanoceria with cerium nitrate (precursor) and urea (fuel), where the resulting cerium oxide nanoparticles were in the form of sphere with the sizes in the range between 10–15 nm.^44^

### 2.4 Decomposition and microwave-assisted heating method

In this method, precursor of nanoparticles are dissolved in organic solvents with the addition of stabilizer, and heated until the decomposition temperature. Stabilizer was added to bind the nucleus of nanoparticles, *i.e.*, it prevents aggregation of nanoparticles. Type of precursors, solvent, stabilizer, reaction temperature and heating time control the quality of nanoparticles. Direct thermal decomposition of cerium(iii) nitrate hexahydrate and ammonium bicarbonate precursors have been carried out without solvents to synthesize nanoceria. After drying at room temperature, the mixture was annealed in a furnace at between 200–300 °C with rate of temperature rise step of 10 °C min^−1^ and 13 nm ceria crystal was produced.^45^ Another precursor, *e.g.*, cerium oxalate decahydrate was also used for synthesizing nanoceria. Dehydration reaction was performed at 150 °C causing anisotropic changes in the crystals dimensions, and fast coarsening of crystallites started at 500 °C. Average size of nanoceria crystallites was between 4–5 nm.^46^ Cerium carbonate hydrate precursors was also used to synthesize spherical nanoceria particles *via* decomposition method in KOH–NaOH molten mixture. CeCO_3_OH was used as an initial material placed in a crucible with a muffle furnace at between 200–700 °C to produce 22–55 nm nanoceria.^47^

Synthesis of dispersible nanoceria was achieved by simple solvent free thermolysis route. The cerium oleate precursor was prepared from cerium nitrate salt and sodium oleate. Cerium oleate powders were heated at 320 °C for different periods of time. The decomposed product was dissolved in hexane and sonicated gently to produce colloidal ceria nanoparticles.^48^ The nanocrystallite cerium oxide powders were also prepared from precursor nanopowders by thermal decomposition. The Ce-propionate powder precursor was prepared by dissolved cerium(iii) acetate sesquihydrate in the methanol : propionic acid (with a 1 : 2 ratio), stirring at 60 °C, then dried at 120 °C in air.^49^

Synthesize of nanoceria by microwave-assisted heating control resulted in high quality of nanoparticles by optimizing parameters reaction. Microwave irradiation could promote synthesis of cerium oxide nanoparticles. He *et al.* compared the characteristics of nanoceria synthesized with cerium nitrate hexahydrate used as precursor in microwave-assisted heating control with traditional thermal methods and. Here, the core of nanoceria had similar crystal structures, where the particle size was slightly smaller. This process is less reactive to variation of redox environment compared to microwave-assisted heating methods.^50^ Microwave-assisted procedure was also carried out to prepare nanoceria with narrow size distribution without any serious agglomeration. In this method, precursors were cerium nitrate, where sodium hydroxide solution were placed in a microwave oven and the end products were dried in a vacuum oven at 60 °C to produce 7 nm nanoceria.^51^

### 2.5 Oxidation and wet synthesis method

**Fig. 1:**
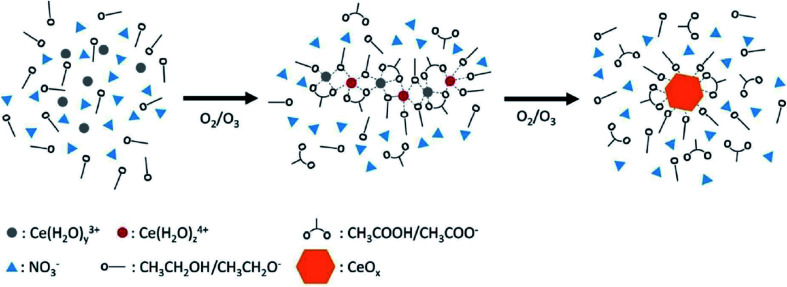
Presentation of a reaction pathway for the synthesis of nanoceria in an ethanolic solution of cerium(iii) nitrate *via* ozonation oxidation. Reprinted from ref. 47 with permission © Royal Society of Chemistry 2020.

Oxidation method is directed using a suitable oxidizing agent to synthesize nanoparticles. In general, this method uses cerium nitrate hexahydrate as precursor, and cerium(iii) ion was oxidized to cerium(iv) using excess hydrogen peroxide (H_2_O_2_) as an oxidizer in acidic or basic aqueous media. Particle size of nanoceria decreases with oxidation concentration. Cerium salts was also used as precursors for preparation of nanoceria *via* wet oxidation synthesize procedures. Cerium(iii) acetate hydrate, ceric ammonium nitrate, cerium chloride heptahydrate, cerium nitrate hexahydrate, and cerium sulphate octahydrate were oxidized with the aid of H_2_O_2_ to form homogeneous dispersion of 100 nm–5 μm crystalline nanoceria.^52^ Oxidation technique and hydroxide-free method were also conducted to synthesize fluorite structured nanoceria using ozone and aliphatic alcohols at room-temperature. Nanoceria with an average size of 2 nm to 4 nm was generated using bubbling ozone into the alcohol cerium salt solutions. Possible pathways for the synthesis of nanoceria could be: (i) oxidation of alcohols into carboxylic acids mediated by ozone and cerium ions, (ii) oxidation of Ce^3+^ to Ce^4+^ by ozone in the presence of acids, and (iii) formation and decomposition of complex polymer network that was made from cerium ions, alcohols, and carboxylates into nanoceria. Fig. 1 shows the schematic of ozonation with ethanolic cerium(iii) solution.^53^

### 2.6 Solvothermal method

Solvothermal method is a synthesis process using reaction in a solvent as liquid (or supercritical) medium at high temperatures. In this method, the solvent, *e.g.*, water or organic compound, is heated until above its boiling point. The use of autoclaves (pressure vessels) is imperative for the reaction to proceed at high temperatures and under high pressures. In one study, nanoceria was prepared with cerium(iii) nitrate as a source of cerium, and deionized water and alcohol to form cerium precursor solution. H_2_O_2_ and ammonia hydroxide were added into the solution, and all precipitates were mixed with anhydrous alcohol, transferred into a teflon-lined autoclave and heated. The method provided 4.8–10 nm nanoceria by filtering and vacuum freeze drying.^54^ A deep eutectic-solvothermal methodology has been developed to synthesize nanostructured ceria at low temperature. Deep eutectic solvents are green solvents that function as an ionic liquid realized by complexing ammonium halide salt with hydrogen bond donor molecules, depressing glass transition temperature (*T*_g_) at the eutectic molar ratio. Hammond *et al.* demonstrated that reline (*e.g.*, choline chloride) combined with urea acts as a latent supramolecular catalyst to bring reactive components together under water, where it acts as a directing agent in deep eutectic solvothermal methods. Reline and its aqueous mixtures are compatible with common ceria precursors, *e.g.*, cerium nitrate hexahydrate or cerium chloride, eliminating the need for high concentration of solubilizing base required in equivalent hydrothermal synthesis. Significant number of short range associated cerium and nitrate ions were observed around choline and urea. Using this method, nanoceria with 5–20 nm size could be achieved.^55^

### 2.7 Green synthesis method

**Fig. 2:**
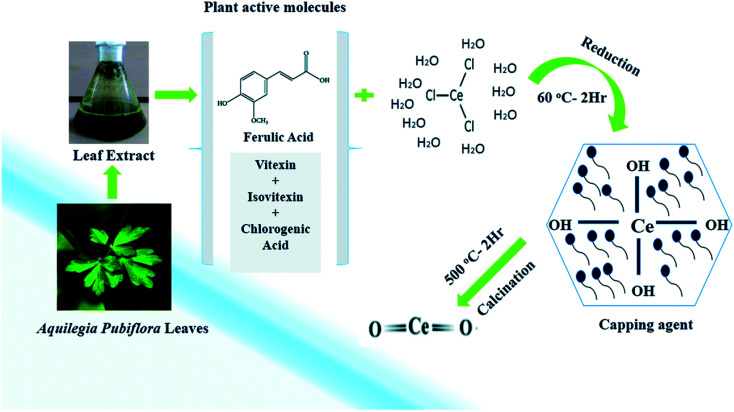
Formation of nanoceria using Aquilegia pubiflora. Reprinted from ref. 51 with permission © Royal Society of Chemistry 2020.

Green synthesis method has recently introduced as an eco-friendly and non-hazardous method (Fig. 2). This approach involves green synthesis of nanoceria, mediated by plants, fungus, nutrients, and biopolymers. Green synthesis of nanoceria with plant mediation has been realized using plant extracts which act as a stabilizing and capping agent. Cerium(iii) chloride heptahydrate (precursor) was added to an aqueous extract of *Aquilegia pubiflora* at 60 °C, centrifuged, oven-dried at 90 °C, and finally calcined at 500 °C. Extract of *A pubiflora* contains phytochemicals, including flavonoids, *e.g.*, vitexin and isovitexin, hydroxycinnamic acid derivatives, *e.g.*, chlorogenic acid and ferulic acid as an effective reducing and capping agent. This approach could yield 28 nm nanoceria. *Pisonia alba* leaf extract with cerium chloride was also used as a capping agent in the green synthesis.^56^

*Linum usitatissimum* seed extract was used for nanoceria synthesis with an aqueous solution of cerium nitrate hexahydrate. Lu seeds contain phytochemical compounds such as phenolic acids, flavonoids, cinnamic acids, and lignin. These components have lone pair electrons that could coordinate cerium atoms as a capping agent.^57^*Salvia macrosiphon* Boiss seed extract was also used for nanoceria synthesis which is rich with flavonoids, *e.g.*, salvigenin, eupatorin, 13-*epi*-manoyl oxide, and sitosterol as capping agents. *Hyphaene thebaica* fruit, *Ceratonia siliqua*, *Clitoria ternatea*, *Azadirachta indica* leaves, *Agathosma betulina* flower, onion extracts, and ripe *Morus nigra* fruit, containing stabilizer and capping agent, were other substances used for nanoceria synthesis.^58–61^ Polyphenolic tannic acid has been introduced as a stabilizing agent for cerium nitrate hexahydrate, with calcination at 400 °C and 600 °C, yielding 7–13 nm nanoceria.^62^ In another work, *Moringa oleifera* leaf was to realize ∼10 nm nanoceria extracted from ammonium ceric nitrate.^63^ Nanoceria, 30 nm in size, was biosynthesized using an aqueous extract of aerial parts of *Prosopis farcta* and cerium nitrate hexahydrate.^64^ Cerium(iv) sulphate was used as precursor in the synthesis using *Ficus carica* leaf extract yielding 10–20 nm nanoceria.^65^ An ultrasound-assisted green synthesis of nanoceria has been reported using a rich piperidine alkaloids leaf extract, *Prosopis juliflora*, with cerium chloride as precursor. Here, the solution was heated at 80 °C, and kept in the microwave oven at 2450 MHz for 10 min, and a yellowish brown precipitate was powdered at 800 °C that yielded 3.7 nm nanoceria.^66^ Khorrami *et al.* used pullulan, a water-soluble polysaccharide that contains maltoriose as a stabilizing agent for nanoceria synthesis. Cerium nitrate hexahydrate solution was added to the pullulan solution at 60 °C, and the obtained resin was heated at 400–600 °C to form nanoceria.^67^ The thermal decomposition of cerium alginate (precursor) was used for the synthesis. Negatively charged centres of alginate chains, *e.g.*, carboxylate groups, bind to trivalent cation of cerium(iii) forming a gel with 3-dimensional crosslinking. This method produces spherical nanoparticles with a size of <5 nm.

Cellulose was also used as a matrix of nano-biocomposites, where nanoceria is embedded in the matrix adopting solution plasma process (SPP), and an eco-friendly synthesis process. SPP-based chitosan-nanoceria does not aggregate or settle down as a pellet when subjected to centrifugation at 10 000 rpm. This approach is very advantageous over the solvent based technique.^68^ In another work, nanoceria was prepared through a sol–gel method using biopolymeric chitosan as an organic template. Cerium nitrate was dispersed in deionized water, and added to the aqueous solution of chitosan. Cerium nitrate and chitosan solutions were mixed, and ammonium hydroxide solutions were added until pH is ∼10, where mixture was stirred at 70 °C until a gel-like material was obtained.^69^ Chitosan-based synthesis was also reported for realizing nanoceria coupled with biocompatible ionic liquid. Here, chitosan was generated by deacetylation process of chitin from the shells of crab. Ionic liquid, 1-butyl-3-methylimidazolium tetrafluoroborate, was used as thermal stabilizer and capping agent as well as surfactant to prevent agglomeration and aggregation of particles, effecting the morphology of nanoceria.^69^ The use of carrageenan hydrogel was used for eco-friendly preparation of nanoceria, where carrageenan forms a bond between polymeric chains to double helix domain through a temperature-induced sol–gel transition, and cerium ions anchor themselves to the –SO_3_^−^ groups into carrageenan. After the gelation process, ceria has few space to escape from polymeric network, and the calcination at temperatures of 400–800 °C yielded nanoceria.^69^

### 2.8 Other synthesis methods

Flame spray pyrolysis method is used to synthesize particles through droplet aerosol formation process. Aerosols are small particles in the form of solids or liquids suspended in gas. Aerosol technology provides an alternative route for ceria synthesize with high surface area at high temperature. Particles in nanometer size are produced with flame spray pyrolysis utilizing atomization or spraying the solution into a flame above an atomization nozzle to produce powdered particles. Spray pyrolysis generally uses three types of atomizer to spray precursor into droplets, *e.g.*, ultrasonic, mechanical, or electrospray. A flame spray pyrolysis method has been introduced to synthesize homogeneous nanoceria from xylene-dissolved liquid precursor, cerium 2-ethylhexanoate.^70^ A thermal treatment technique has been introduced which uses polyvinylpyrrolidone (capping agent) and deionized water (solvent), where calcination treatment was carried out at 500–650 °C to crystallize nanoceria. In this method, cerium nitrate hexahydrate was used as the precursor material.^71^ A modified polymer complex (PC) process was used to obtain nanoceria with high purity at low temperature. PC method successfully reduces individualities of different metal ions, achieved by encircling stable metal–chelate complexes *via* a growing polymer net. This rigid organic polymer net reduces segregation of particulate metals during the decomposition process of the polymer.^72^

Reverse micelle is another synthesis method, where cerium nitrate hexahydrate solution was mixed with colloidal micelle solution. Ammonium hydroxide solution was added into the mixture, and incubated until nanoceria slowly formed in the reversed micelles. Cerium oxide nanoparticles were rinsed with ethanol and water, centrifuged, collected, and dried at 50–60 °C.^73^ Aqua phase nanoceria synthesis was performed *via* 6-aminohexanoic acid (6-AHA). Cerium(iii) nitrate solution was added continuously into an aqueous solution containing 6-AHA by using a syringe pump at 95 °C. The 2D self-organization ceria nanosheets were formed, which initially formed small ceria nanocrystals followed by an *in situ* recrystallization process.^74^ Colloidal synthesis strategy has developed for synthesis of ceria nanocubes with controlled morphology. Cerium nitrate, ammonium cerium nitrate, ammonia solution, and ammonium acetate are used as reactants operated under 80 °C. Existence of acetate radical ions is the critical factor in the formation of nanocubes ceria.^75^ Surfactant free-aerogel synthesis involves the preparation of sols of metal salts followed by gelation, and finally the exclusion of solvent by air to produce nanoceria. In this method, solvent can be excluded from gel by simple drying. In this method, nanoceria is produced with lesser capillary action, pore collapsing, low density, and high-surface-area. Cerium(iii) nitrate hexahydrate and oxalic acid (precursor) and toluene–ethanol (solvent) mixture were used to obtain 3–5 nm nanoceria in size.^76^ Sol–gel method uses different solvents such as water, acetone, ethanol, and ethylene glycols, with cerium(iii) nitrate hexahydrate, where ammonium hydroxide is used as precursors.^77^

## 3 Characterization studies

Material characterization is a method for obtaining information on structure, composition, and defects of materials. Characterization provides information on the physical and chemical properties of the nanomaterials. Morphologies (size, shape and distribution) of nanoceria can be characterized by spectroscopic, microscopic, X-ray technique, and thermal stability methods. It is still challenging to determine physicochemical properties of nanoparticles and to explore their structure–function relationships due to the fact that the nanoparticles are prone to agglomeration as well as they could have a broad distribution of sizes, shapes, and defects. These characteristics require comprehensive analysis of nanoparticle behavior. One important issue to consider is that the characterization methods can directly affect the measured nanoparticle quantities.

### 3.1 UV-visible spectrophotometry

**Fig. 3:**
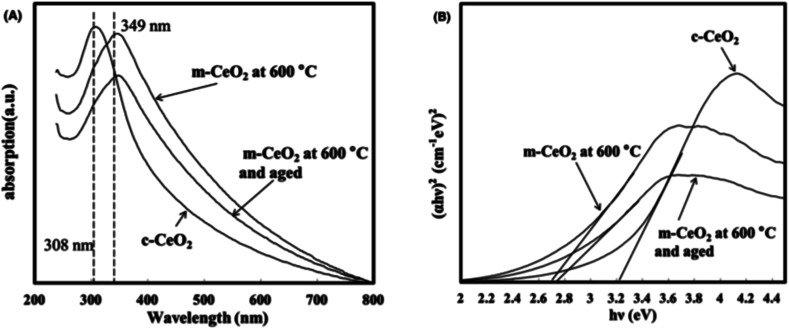
(A) UV-Visible spectra of commercial nanoceria (c-CeO_2_), molten salt nanoceria synthesized (m-CeO_2_) at 600 °C, and m-CeO_2_ aged, and (B) the dependence of (*αhν*)^2^ on photon energy (*hν*). Reprinted from ref. 75 with permission © 2017 The American Ceramic Society.

UV-Visible characterization could be carried out to determine optical properties and the band gap energy of synthetized nanoceria. Absorption of nanoceria in the UV region arises due to the charge transition between O (2p) and Ce (4f) expressed in O^2−^ and Ce^4+^. In general, maximum absorption peak for nanoceria is observed between 290 and 360 nm. Moreover, maximum absorption peak of nanoceria shifted towards lower wavelengths, which is associated with the quantum confinement effect due to the decrease in particle size. Band gap energy incorporated with the transition between valence and conduction band in ceria. Absorption of photons from UV-Vis spectrophotometry depends on the nature of the semiconductor material, and the wavelength of the incoming light. Absorption of a semiconductor material causes the excitation of electrons from the valence band to the conduction band. The band gap energy can be determined by Tauc plot method. The decrease in band gap energy can indicate larger nanoparticle sizes due to the increase in the crystallinity of nuclei CeO_2_ which forms a cluster, and becomes a dense and regular crystallite grain.^78,79^ Molten salt based methods were also developed using KCl–LiCl to synthesize polyhedral nanoceria. Crystal structure of Ce_2_(CO_3_)_3_·*x*H_2_O broke down to crystal nuclei of nanoceria in the molten salt system. Fig. 3 shows the UV-Visible spectra of KCl–LiCl salt system of nanoceria. Absorption peak of commercial nanoceria (c-CeO_2_) was centered at 308 nm, whereas the molten salt product (m-CeO_2_) was found at 349 nm. A significant red shift in the spectrum were observed, m-CeO_2_'s aged powder were found. Here, particle shape and the level of cerium species contribute these red or blue shift in the UV-Visible spectra, *e.g.*, blue shift is due to the decrease in Ce^4+^ after the reduction reaction, while the red shift is due to the increase in Ce^4+^ after oxidation reaction. Lower band gap energy of m-corresponded to the lower of crystallinity 111 (69.7%) and the higher strain percentage (0.589%) of m-CeO compared to c-CeO_2_ (79.8 and 0.389%). Different band gap also corresponded to the electronic transition, and depended on the methods of synthesis and particle size.^80^ The effect of calcined temperatures of nanoceria synthesis resulted in a red shift in the UV-Visible spectra. This phenomenon corresponded to the increase in annealing temperature enhancing the crystalline properties. UV-Vis absorption ability of crystalline nanoceria depends on the band gap energy, *e.g.*, high band gap energy enhances the interactions for smaller nanoparticles.^44^

### 3.2 Fourier transform infrared (FTIR) spectroscopy

**Fig. 4:**
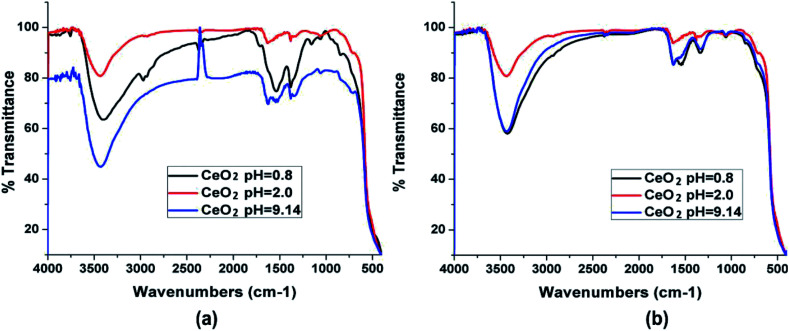
IR spectra of nanoceria powders at different pH values (a) without heat treatment and (b) treated at 550 °C. Reprinted from ref. 76 with permission © 2020 Elsevier B.V.

FTIR spectroscopy is a strong tool to identify different types of molecular bonds and functional groups, and to study the vibrational motion of atoms and molecules. Here, the spectrum peak at the frequency between 450 and 700 cm^−1^ corresponded to the O–Ce–O-vibration. Residual water and hydroxyl groups are detected in synthesized nanoceria (stretching vibration) at 1600, 1300, and between 3200 and 3400 cm^−1^. Wide bands at 1000, and between 1300 and 1400 cm^−1^ are related to the formation of carbonate-like groups on CeO_2_ surface.^37,79^ Effect of calcination temperature (100 °C and 200 °C) of the synthesized nanoceria resulted in large IR absorption band at 500 cm^−1^ due to Ce–O bond tension. In addition, the presence of CeO_2_ in the un-calcined specimen indicated by an absorption band at 840 cm^−1^ is a typical Ce–O stretching vibration. Peaks are observed at 1020, 1050, and 1100 cm^−1^ corresponding to calcined samples due to the interaction of cerium with CO_2_ resulting in Ce–O–C bonds stretching vibrations.^29^Fig. 4 shows the IR spectra for nanoceria powders. The nanoceria was obtained at different pH values with pre-calcined at 350 °C, and with heat treatment at 550 °C. Vibrational modes of Ce–O showed large band spectra between 400 and 630 cm^−1^. A broad band at 3450 cm^−1^ was associated with stretching vibration of O–H corresponding to the residual water or hydroxyl groups. The bands at 1300 and 950 cm^−1^ corresponded to the vibrational tension of H_2_O. The variations in intensity at ∼1625 cm^−1^ is associated with H–OH flexion that overlaps with the band corresponding to O–C–O stretching, following calcination.^81^

### 3.3 Raman spectroscopy

Raman spectroscopy is widely used method for studying structures, crystal defect, and films deposited on a particular substrate. In general, cerium oxide has a fluorite structure, which generates three peak frequencies, *e.g.*, 272 cm^−1^ and 465 cm^−1^ corresponding to doubly and triply degenerate transverse optical mode, and 595 cm^−1^ related to non-generate longitudinal optical mode. Symmetric stretching at 460 cm^−1^ of Ce–O incorporated with F2g vibrational mode of crystalline cubic fluorite type of ceria.^82,83^ Effect of pH of nanoceria on the Raman spectra has been described.^72^ The spectrum obtained at pH = 9.1 showed bands at 1315, 1430, 1483, and 1561 cm^−1^. The bands between 1300 and 1400, and at 1560 cm^−1^ was associated with adsorbed O_2_. The system contained large amounts of carbon which favored reducing atmosphere, and thus the presence of Ce^3+^ below 550 °C during the synthesis of ceria. Carbon monoxide (CO) and carbon dioxide (CO_2_) can be also absorbed in the range between 1700 and 1800 cm^−1^ (bridged carbonate) and 1430 and 483 cm^−1^ (unidentate carbonate). Raman spectra were recorded between 410 and 520 cm^−1^, and between 1000 and 1400 cm^−1^ to show the effect of heat treatments on ceria samples synthesized at pH = 2.

**Fig. 5:**
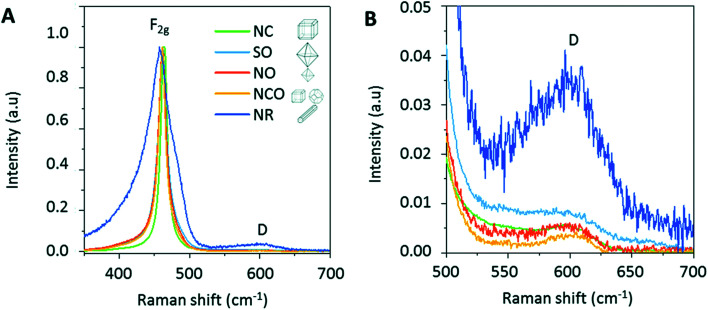
(A) Raman spectra of CeO_2_ particles recorded in between 350 and 700 cm^−1^, and (B) between 500 and 700 cm^−1^, where excitation source is at 633 nm. Reprinted from ref. 77 with permission © Royal Society of Chemistry 2020.

In these spectra, amplitude and the spectral position of the bands at ∼460 cm^−1^ (symmetric mode of Raman vibration F2g), and at 1160 cm^−1^ (characteristic of the surface superoxide species, O_2_^−^) changed. Fig. 5 shows the Raman spectra of the nanocubes (NC), nanorods (NR), and nano-octahedra (NO), submicronic octahedral (SO), and nanocubes + nanotruncated octahedral (NCO) of different sizes from hydrothermal process, in the range between Raman shift 350 and 700 cm^−1^. The strong peak at 460 cm^−1^ due to the CeO_2_ fluorite phase associated with a symmetric breathing of the oxygen atoms around cerium ions. This mode is size dependent and effected by irregularities in the sub lattice. This fact is shown in Fig. 5A, where F 2g peak of the nanorods is broader compared to other morphologies. The weak peak around 600 cm^−1^ (denoted with D) shown in Fig. 5B was associated with oxygen defects, and due to the presence of Frenkel-type oxygen vacancies were an oxygen atom displaced from its lattice position to an interstitial site.^82^

### 3.4 Photoluminescence spectra

Photoluminescence (PL) is a photo induced fluorescence technique to investigate energy levels of materials. Violet emission peak at 477 nm and the green emission at 508 nm correspond to the surface defects. Multiple studies have been reported showing that these peaks are associated with displaces or oxygen defects that contribute to oxygen vacancies at nanoceria. Electrons excited from the valence band to the 4f band of the oxide act as defects. The reduction in the O_2_ concentration increasing oxygen partial pressure resulted in the decrease within the peak intensity of PL spectra.^84^

### 3.5 Scanning and transmission electron microscopy

Scanning electron microscopy (SEM), field emission scanning electron microscopy (FESEM), transmission electron microscopy (TEM) are the fundamental tools to determine morphology and size of the nanoparticles that results in image contrasts of the samples in nanoscale. SEM and TEM incorporated with energy dispersive X-ray spectroscopy (EDS or EDX) can provide elemental composition information of samples.

**Fig. 6:**
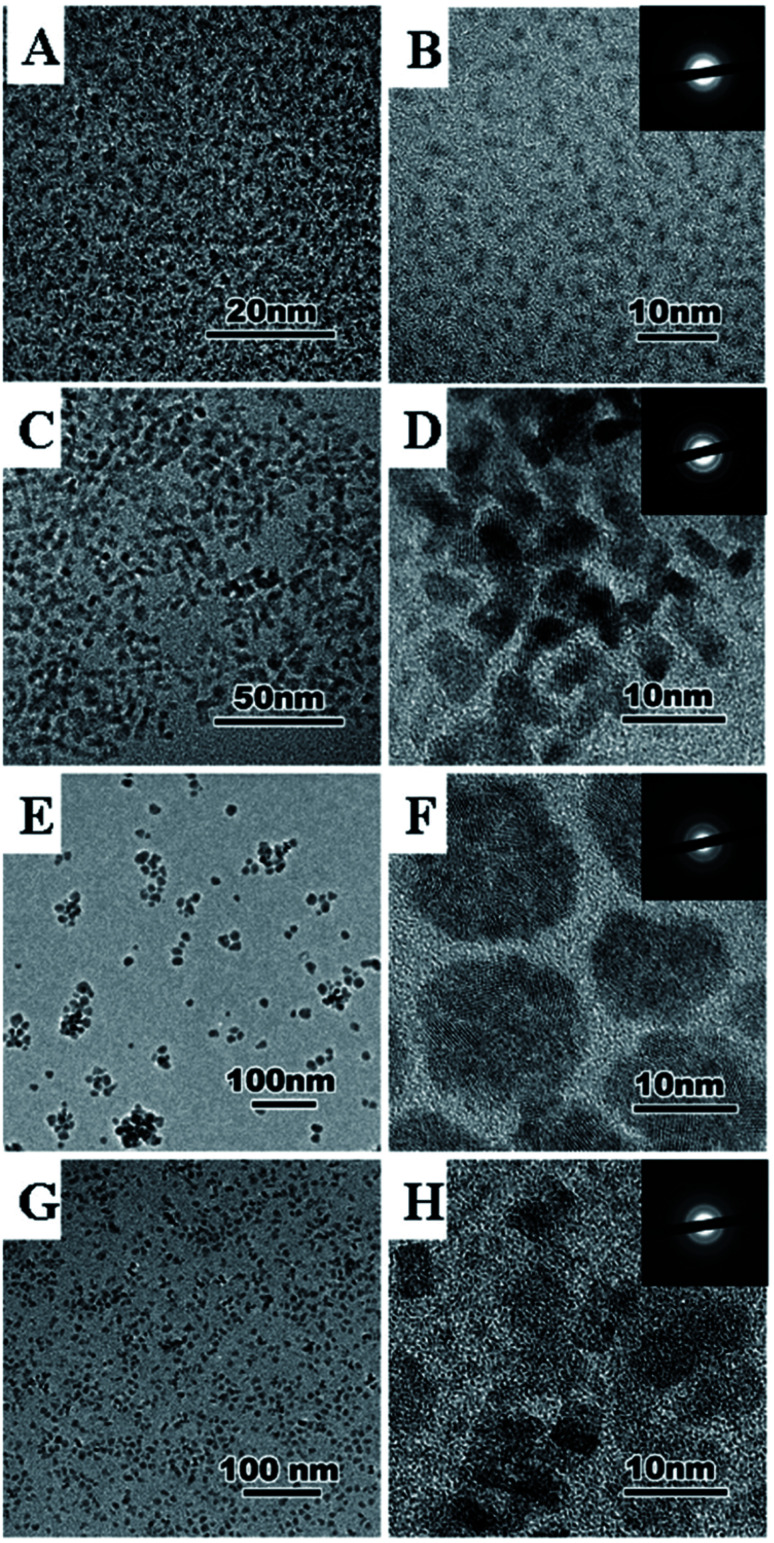
TEM images of ceria nanoparticles synthesized by thermolysis of cerium oleate at 0.3 mbar and 320 °C for different reaction times: 0.7 h, 0.3 mbar (A and B), 24 h, 0.3 mbar (C and D), 72 h, 0.3 mbar (E and F), 144 h, 0.3 mbar (G and H). Reprinted from ref. 42 with permission © Royal Society of Chemistry 2020.

Here, the samples are shot with a high-energy electron beam which hit the sample, get reflected, and read by a detector.^29,34^ SEM and TEM images can distinguish agglomeration or decomposition of nanoparticle clusters. Fig. 6 shows representative TEM images for the nanoceria synthesized by thermolysis of cerium oleate at 0.3 mbar and 320 °C for different times. Decomposition products of nanoceria based on decomposition time with different shapes and sizes were depicted clearly. Here, morphology (size, shape, distribution) of nanoceria growth is built by a nucleation dissolution recrystallization step mechanism, depending on the reaction time.^48^Fig. 6A and B show nanoceria with average size of 2 nm formed 0.7 h decomposition time. Fig. 6C and D show 8 nm rectangular single-crystal nanoceria for the decomposition time of 24 h. Formation of 15 nm polycrystalline spherical nanoceria could be seen after the decomposition time increased to 72 h (Fig. 6E and F). 4 nm single crystalline nanoceria was obtained after 144 h.

### 3.6 X-ray diffraction

X-ray diffraction (XRD) is the method used to determine structure and the crystalline size of nanoceria. Average nano-crystalline size (*D*) is calculated using the Scherrer formula; *D* = *Kλ*/*β* cos *θ,* where *D* is the average crystallite size, *K* is Scherer constant (0.9), *λ* is the wavelength of the incident X-rays, *β* = √(*B*_2_ − *b*_2_), where *β* is the full width at half maximum (FWHM) after correcting for instrumental broadening, *B* is the observed FWHM of the film, *b* is the instrumental broadening, and *θ* is the diffraction angle.^83^

**Fig. 7:**
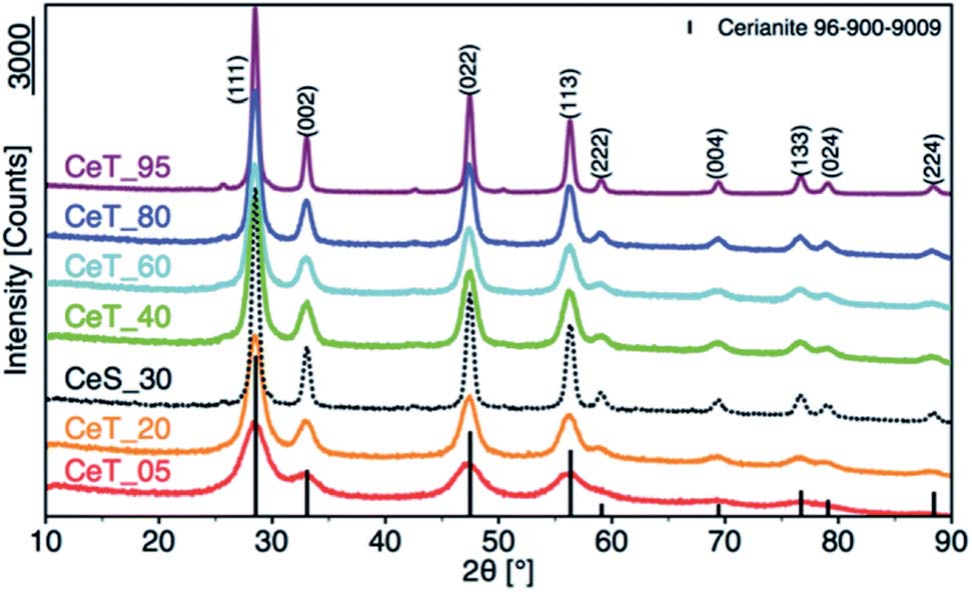
XRD pattern of nanoceria synthesized at different reaction temperatures, *e.g.*, 5, 20, 30, 40, 60, 80, and 95 °C. Reprinted from ref. 80 with permission © The Royal Society of Chemistry 2020.

Fig. 7 illustrates the XRD pattern of ceria nanoparticles in the range of angle 2*θ* between 5 °and 80°. Nanoceria was synthesized by a low temperature water-based precipitation method. (111), (200), (220), (311), (222), (400), (311), and (331) peaks were obtained for the desired temperatures. These peaks are indexed to a pure cubic phase of CeO_2−*x*_ (0 < *x* < 0.5) with lattice constant *a* = 5.420 Å, according to The Joint Committee on Powder Diffraction Standards (JCPDS file no. 34-0394, space group *Fm*3*m*). XRD patterns show four main peaks, *e.g.*, (111), (200), (220), and (311) of nanoceria that are well-defined and indicated correspond to the fluorite-type structure. The broadened peak corresponded to the nanometer-sized crystallites. The average crystallite size calculated with Scherer equation was found as between 2 and 15 nm.^85^

### 3.7 X-ray photoelectron spectroscopy

**Fig. 8:**
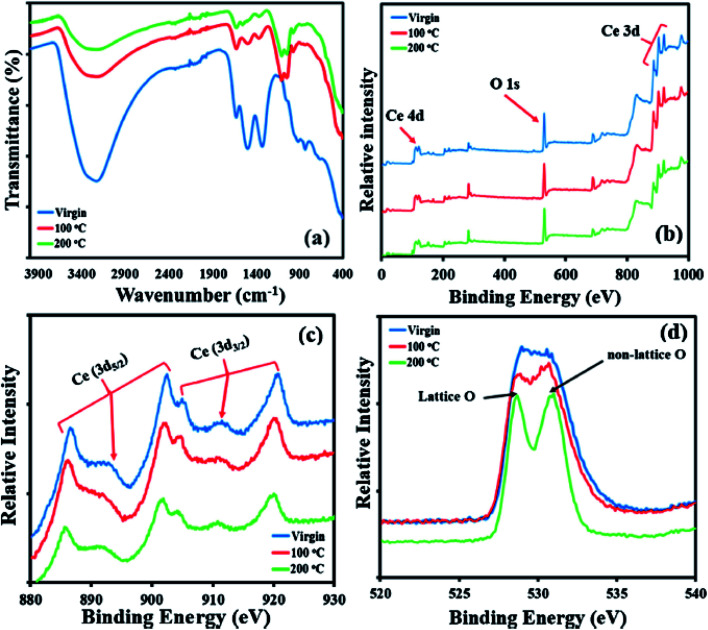
Chemical composition of un-calcined and calcined ceria nanoparticles (a and b) using FTIR, and (c and d) and XPS. (a) FTIR spectra for all samples showing the presence of major Ce–O bonding peaks. (b) XPS scans showing the presence of Ce and O, with small amounts of C due to atmospheric contamination. (c) High-resolution scans for cerium showing the peaks belonging to the oxidation state of 4+. (d) Peaks for lattice oxygen and non-lattice oxygen that are visible at 529 and 531 eV respectively. Reprinted from ref. 29 with permission © 2020 Elsevier B.V.

X-ray photoelectron spectroscopy (XPS) is a non-destructive technique to investigate chemical and electronic structure of materials, and to characterize surface elemental composition. An important advantage of XPS is its ability to identify binding energies of the chemical shifts of elements, *e.g.*, the shifts associated with the oxidation state of cerium in nanoceria. Matin *et al.* described XPS analysis of un-calcined and calcined precipitations nanoceria synthesis method. In this study, glass slides were coated with ceria particles, and a range of energies between 0 and 1000 eV were used for identifying elements based on the binding energy survey scans. Next, high resolution scans were carried out to provide further details of lattice position and oxidation states. XPS spectra can exhibit the crystal structure of solid, *e.g.*, a cubic, fluorite type for Ce^4+^ in CeO_2_, or a hexagonal structure such as sesquioxide type for Ce^3+^ in Ce_2_O_3_. Fig. 8 shows the surface elemental analysis using XPS for un-calcined and calcined nanoceria. Fig. 8a shows the FTIR spectra of the synthesized nanoparticles before and after the calcination at 100 °C and 200 °C. Fig. 8b and c shows the photoelectron peaks of cerium, *e.g.*, Ce 3d (∼900 eV) and Ce 4d (110 eV), while O 1s for oxygen due to the presence of elements detected on the surface of the coated substrates. In addition, C 1s ∼285 eV confirmed the atmospheric contamination such as carbon that can interact with surface oxygen to form carbonates. Here, the peaks are also associated with the oxidation states, Ce^3+^, Ce^4+^, and lattice and non-lattice of oxygen. Fig. 8c also shows the minor differences in the spectra that are associated with temperature of calcination of nanoceria. Higher temperatures increase the proportion of carbon due to the contamination of hydrocarbons on the surface. Fig. 8d presents XPS high resolution scans for oxygen broken down into the lattice structure (∼529 eV) at their regular site in nanoceria, and non-lattice oxygen atom (∼531 eV) adsorbed onto the surface, *e.g.*, hydroxyls or carbonates.^29^

### 3.8 Thermogravimetric analysis

**Fig. 9:**
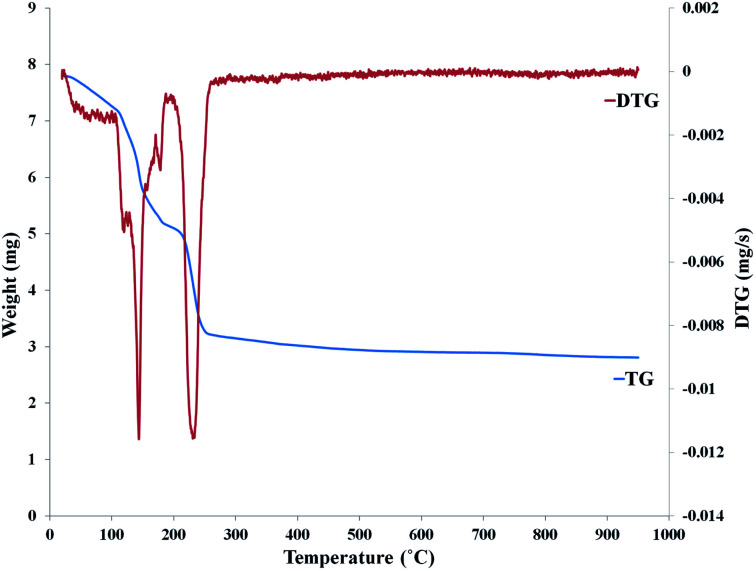
TGA and DTG diagrams of nanoceria that was prepared with 30 mL Salvia macrosiphon Boiss seed extract. Reprinted from ref. 81 with permission © 2020 Elsevier B.V.

Thermogravimetric analysis (TGA) is a technique for studying thermal behavior of the samples based on changes in chemical and physical properties in response to the changes of temperature. The change in sample mass at TGA is indicated as a function of temperature and time. Another thermal analysis is the differential thermal analysis (DTA) that measures the temperature difference, Δ*T*, between the temperature of sample and reference material. Modern and automatic thermal analysis equipment makes it possible to carry out simultaneous TGA and DTA measurements. Fig. 9 shows the TGA and DTG diagram of the nanoceria prepared from *Saliva macrosiphon* Boiss seeds (SmB) extract which is a capping agent. TG/DTG analysis was used to evaluate thermal behavior of nanoceria. The peak centered at 145 °C in the DTG curve confirmed the first 34% weight loss, and is related to the reduced water absorption on the surface and pore nanoceria. TGA curve peak centered at 230 °C in DTG curve was related to the 30% weight loss between 200 and 400 °C, and decomposition of the organic compound of SmB seeds extract. 64% of total weight loss up to 400 °C, and there was no thermal feature was observed above 400 °C which confirmed its thermal stability up to 1000 °C.^86^

## 4 Applications of nanoceria in electrochemical sensors

Nanoceria is widely used in electrochemical sensing with better reactivity due to their crystal structure with multiple oxygen vacancy defects, and high ionic conductivity characteristics. Unique characteristics of ceria and nanoceria, *e.g.*, catalytic activity, enzyme mimetic properties, ability to transfer oxygen, switchable redox reactivity, surface coating, and surface reactivity, allow their use in electrochemical sensors. Their high isoelectric point, adsorption capacity, mechanical strength, non-toxicity, and good biocompatibility, enable them to adapt to the requirements for sensors employed with biological matrices.^87^ Nanoceria is an ideal material to immobilize negatively charged biomolecules, due to its isoelectric point, *e.g.*, 9.2. These nanoparticles can carry our different functions in sensors, *e.g.*, transduction element, amplifier to enhance chemical and electrochemical signals, catalyst and nanozyme that can replace biological enzymes, and label in bio-affinity assays.^88,89^ Electrochemical behavior of ceria and nanoceria depend on their morphologies affected by the growth rates of ceria nanocrystalline relied on solvent composition. Furthermore, shifting between oxidation states of Ce^3+^ and Ce^4+^ enhances electrochemical property of nanoceria.

### 4.1 Electrochemical sensor

Electrochemical sensors consist of two basic components, *e.g.*, a chemical recognition system and a physical transducer. Physical transducers are electrodes that convert recognition system to a measured electroanalytical signal. The use of nanoparticles in electrodes one strategy to increase sensitivity, to enhance stability, and to improve performance of electrochemical methods. In most cases, the presence of ceria increase the surface area, *i.e.*, more nanoparticles could attach to the surface which enhances the direct electron transfer rate.^89^ For example in one study using nanoceria, a non-enzymatic sensor for monitoring glucose was developed. The combination of nanoceria and gold nanoparticles (AuNPs) on glassy carbon electrodes (GCE) enhanced the enzyme–electron transfer, and increased the electron transfer rate. Surface of GCE was modified by dropping and drying dispersion of CeO_2_, followed by addition of chitosan to fix CeO_2_ nano crystalline. CeO_2_ modified electrodes were subsequently immersed in tetra chloroauric acid (HAuCl_4_) solution. Then, the constant potential was applied over the time to deposit AuNPs to the electrode surfaces. Detection limit was found as 2.86 × 10^−3^ mM with a linear range between 0.02 and 0.6 mM. The sensor also showed good stability, reproducibility, and selectivity.^90^ C/CeO_2_ composites also showed an excellent performance as an electrochemical glucose sensor. Composites were attached to GCE surface using chitosan (C). C/CeO_2_ coated electrodes detected glucose in the range between 2.0 μM and 1.8 mM with a detection limit of 0.8 μM.^91^

**Fig. 10:**
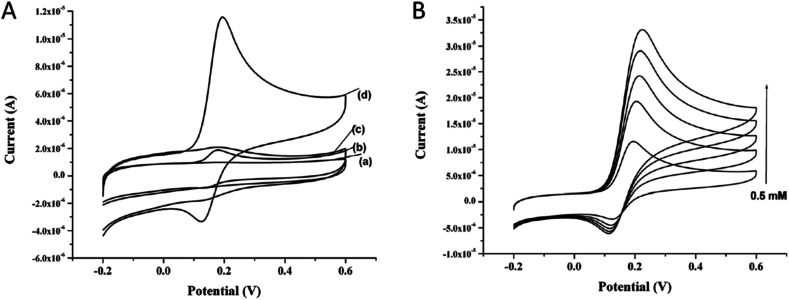
(A) Cyclic voltammograms of (a) bare CPE, (b) CPE + dopamine, (c) CPE/nanoceria, and (d) CPE/nanoceria + dopamine. (B) Cyclic voltammograms of CPE/nanoceria modified electrodes with various concentration of dopamine from 0.5 to 0.25 mM. Reprinted from ref. 88 with permission © 2020 Springer Nature Switzerland AG.

Carbon paste electrode (CPE) modified with CeO_2_ nanorods embedded in nickel hydroxide (Ni(OH)_2_) matrix was used for non-enzymatic glucose detection. Electrochemical response of CeO_2_/Ni(OH)_2_ nanocomposite was significantly improved due to the synergetic effect between CeO_2_ and Ni(OH)_2_. LOD was found as 1.13 μM, and response time was less than 5 s.^92^ The affinity of CeO_2_ towards H_2_O_2_. Using this method, direct measurement of H_2_O_2_ could be used to detect, quantify, and diagnose pathological conditions, *e.g.*, infection and inflammation.^89^ Modified GCE *via* nanohybrid of single-walled carbon nanohorns (SWCNHs) enveloped with CeO_2_ in a core–shell hierarchy was successfully applied as an electrochemical sensor. Ink-like suspensions of SWCNHs@CeO_2_ were cast on the clean GCE surface, and dried in the oven. Electro-reduction of H_2_O_2_ was catalyzed with the fast transition between Ce^3+^ and Ce^4+^ that changed the number of oxygen vacancies due to the synergetic effect of CeO_2_ layer and the nanocarbon. The constructed biosensor was selective, robust, and anti-interference of some materials.^92^ Electrochemical sensors based on nanoceria for the detection of dopamine (DA) molecules were also reported. Therefore, the detection of dopamine with high sensitivity is quite essential. Fig. 10A presents the cyclic voltammograms of bare and nanoceria modified CPE in the presence or absence of DA. Bare CPE showed a small DA oxidation peak at +0.2 V (Fig. 10A(a)). On the other hand, nanoceria modified CPE showed a well-defined and strong oxidation peak at +0.2 V, and a reduction peak around +0.13 V (Fig. 10A(c)). Increasing DA concentration resulted in an increase in the anodic peak current as shown in Fig. 10B. This data confirmed that the surface confined species resulted in a redox peak current.^93^

**Fig. 11:**
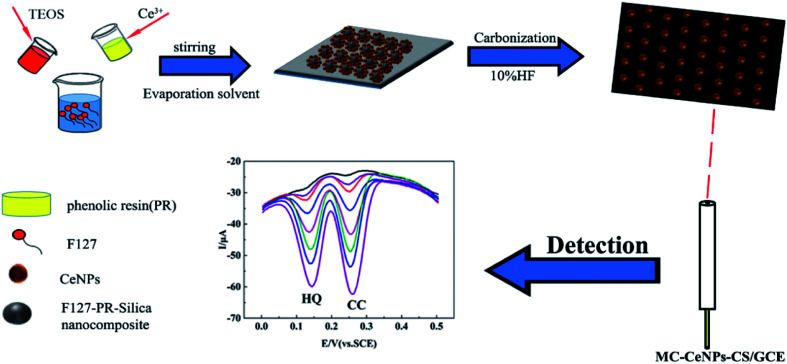
Scheme of the fabrication of mesoporous carbon and ceria nanoparticles composite (MC–CeNPs), and the simultaneous detection of hydroquinone (HQ) and catechol (CC). Reprinted from ref. 93 with permission © 2019 MDPI, Basel, Switzerland.

Nanoceria can neutralize free radical chemical species by undergoing redox changes (Ce^3+^ ↔  Ce^4+^). Based on this characteristic, cyclic voltammetry (CV) and chronoamperometry responses of multi-valent nanoceria on the GCE in presence of H_2_O_2_ was developed as an enzyme-free nanoceria-based sensor. This electrochemical sensor detected ultra-low concentration of analyte, *e.g.*, limit of quantitation is 0.1 pM of H_2_O_2_.^94^ Michaelis–Menten mechanism of catalase-like activity of ceria film on GCE electrode was developed for H_2_O_2_ detection below 5 μM detection limit without using a mediator.^95^ In a recent study, multi walled carbon nanotubes (MWCNTs) with nanoceria and poly-3,4-ethylenedioxythiophene (MWCNTs/CeO_2_-PEDOT) modified GCE was used for their electrocatalytic effects toward dopamine. Under optimum conditions, two wide linear ranges, 0.1–10 μmol L^−1^ and 40–400 μmol L^−1^ with a low limit of detection, *e.g.*, 0.03 μmol L^−1^ was obtained. Interferences were not observed for the designed sensor.^96^ Detections of DA, ascorbic acid, uric acid, and acetaminophen were studied using CeO_2_-carbon nanotubes (CNTs) modified GCEs. The existence of nanoceria on CNT surface increased the surface area and electron transfer between neuro-transmitters and electrodes. Electrochemical behavior of electrodes was determined with differential pulse voltammetry (DPV), electrochemical impedance spectroscopy (EIS), and CV. A linear behavior was determined for DA, ascorbic acid, uric acid, and acetaminophen have been ranged 0.01–900 μM, 0.01–700 μM, 0.01–900 μM, and 0.01–900 μM with detection limits of 3.1 nM, 2.6 nM, 2.4 nM and 4.4 nM, respectively.^97^ A mesoporous carbon and nanoceria composites (MC–CeNPs) modified GCEs were developed to detect hydroquinone (HQ) and catechol (CC) with high sensitivity and selectivity. Attaching (MC–CeNPs) onto electrodes resulted in fast electron transfer ability. Fig. 11 shows the schematic of the preparation of MC–CeNPs modified electrodes.^98^ Screen printed carbon electrode (SPCE) modified with polyacrylic acid coated nanoceria (PAA–CNPs) was developed based on Fe(CN)_6_^3−/4−^ redox system. Deposition of PAA–CNPs into SPCE was carried out by applying a potential. Here, the presence of PAA–CNPs on the electrode surface exerted a high oxidative effect on Fe(CN)_6_^4−^.^99^ The gold electrode (GE) modified with ceria microfluidic sensor was also developed for the detection of H_2_O_2_ directly secreted from living cells. CeO_2_ nanosheets exhibited a triple-enzyme mimetic activity (oxidase, catalase, and peroxidase-like on GE), *i.e.*, H_2_O_2_ in the living cell could be detected. The sensor showed a high sensitivity threshold 226.4 μA cm^−2^ μM^−1^ with a low detection limit of 20 nM.^100^

CeO_2_/reduced graphene oxide (rGO) nanocomposite modified GCEs have been constructed for the detection of DA and ascorbic acid. rGO was used as a matrix in the composite, increased the concentration of oxygen vacancies, coupled and exhibited the improvement of the nanocrystal conductivity and charge transfer (Murali *et al.* 2019). CeO_2_/rGO–GCE showed a good performance and selectivity for the detection of organophosphorus pesticide fenitrothion, even in the presence of interferences in water samples.^101^ CeO_2_/graphene oxide (GO) composite-based electrochemical sensor was developed for the detection of toxic methyl orange as a dye. GCE was modified with GO-polylactic acid (PLA)–CeO_2_, and the electrochemical characterization of GO–PLA–CeO_2_ composite exhibited a very advantageous behavior for dye removal.^102^ GCE modified with organic–inorganic nil-blue-CeO_2_ nanohybrid was developed for the detection of hydrazine.^103^ A nanostructured copper–ceria (CuO–CeO_2_) composite prepared by calcination of Cu(ii)/Ce(iii) metal organic framework was developed for the detection of insecticide malathion.^104^ An amperometric sensor which contained CeO_2_–CuO modified GCEs was designed for nitrite detection.^105^ Nanoceria as nanocomposite with Cu_2_O and platinum was introduced for a sensitive electro-oxidation and selectivity capacity toward the detection of DA and paracetamol. Pt/CeO_2_@Cu_2_O modified CPE exhibited a linear behavior for DA and paracetamol with the detection limits, *e.g.*, 0.079 μM and 0.091 μM, over the range between 0.5 and 100 μM.^106^ The stannum doped CeNPs modified glassy carbon paste electrode was used for the detection of an anti-cancer drug, *e.g.*, dacarbazine.^107^ In this work, ruthenium-loaded CeO_2_ nanocubes (Ru/CeO_2_) with rich oxygen vacancies were used to construct an electrochemical sensing interface. The designed nanocomposite electrode enhanced the electrochemical signals, and enabled the detection of Hg(ii) ion.^108^ Fe_3_O_4_/CeO_2_@Au modified GCEs was developed for microRNA-21 detection, and exhibited good repeatability, stability, reproducibility, and anti-interference ability.^109^

**Fig. 12:**
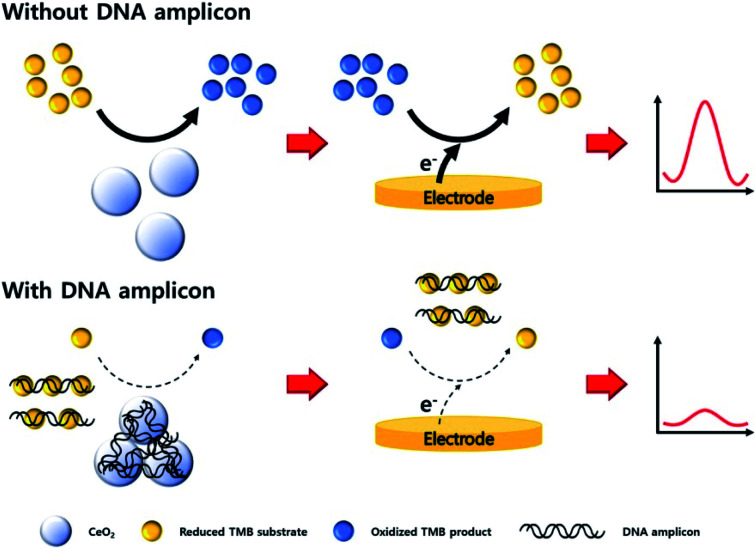
Schematic illustration of the DNA detection with an electrochemical method based on oxidase-mimicking activity of CeO_2_. Reprinted from ref. 107 with permission © 2020 Elsevier B.V.

Modifications on the screen printed electrodes (SPEs) with nanoceria was developed. A colloidal nanoceria was dropped onto SPEs. SPEs/CeNPs was used in the detection of total antioxidant capacity, *e.g.*, caffeic acid, gallic acid, quercetin, ascorbic acid, trans resveratrol, and dimethyl sulfoxide in wine samples.^110^ Electrochemical sensor for the detection of oxymetazoline hydrochloride was developed by modifying SPEs with nanoceria. The developed sensor used paraffin oil (pasting liquid) and nanoceria (conductor) to increase electrical conductivity of electrode surface area. An ion pairing agent, *e.g.*, tetraphenyl borate, was incorporated with the sensor as a sensing membrane of the drug material. SPEs/CeNPs sensor displayed a good performance, significant linear response, and stable reproducible potential for five months.^111^ GE was modified with nanoceria, and showed oxidase-mimicking activity with 3,3′,5,5′-tetramethylbenzidine (TMB) substrate. Oxidase-like reaction of nanoceria-TMB complex could carry a DNA amplicon that changes the electrochemical signal. The proposed sensor exhibited a rapid determination of *E. coli* DNA amplicon without the need for a post-purification. Fig. 12 shows the schematic illustration of the electrochemical DNA detection based on oxidase-mimicking of nanoceria.^112^

Another nanoceria based electrochemical sensor for H_2_O_2_ detection was developed based on the formation of nanocomposite gold nanoparticles-graphene sheets@cerium oxide (GS@CeO_2_) modified GE. GEǀGS@CeO_2_/Au showed a good performance towards the electrocatalytic reduction of H_2_O_2_, and achieved a low limit of detection of 2.6 × 10^−4^ mM.^113^ The screen printed nanostructured cerium oxide was applied on pre-fabricated GE for the indirect detection of melamine in the presence of ascorbic acid. A wide linear melamine concentration range between 0.01 ppb and 10 ppm, and the limit of detection without and with ascorbic acid, *e.g.*, 1.5 × 10^−3^ and 2.4 × 10^−3^ ppb, respectively were obtained by using this electrode.^114^ The use of nanoceria with different electrode types, and modification techniques for various analytes introduced in the last four years were summarized in Table 1.

**Table 1:** Application of nanoceria in electrochemical sensing in the last four years (2017–2020). Abbreviations: LoD: limit of detection, LR: linear range, S: sensitivity

Electrode	Electrode modification	Target	Synthesis	Characterization	Characteristic	Ref.
GCE	GCE/CeO_2_/chitosan-Au	Glucose	—	SEM, EDS, XRD, TEM	LoD: 2.86 × 10^−3^ mM	90
					LR: 0.02–0.6 mM	
	GCE–C/CeO_2_ composites-chitosan	Glucose	Solvothermal	XRD, EDS, SEM, TEM, XPS	LoD: 0.8 mM	91
					LR: 2.0 mM–1.8 mM	
	GCE/SWCNHs@CeO_2_	H_2_O_2_	Sol–gel	TEM, AFM, TGA, FTIR, Raman, EDX, electrochemistry	S: 160 μA cm^−2^ mM^−1^	92
	GCE–CeO_2_	H_2_O_2_	—	XPS, SEM, AFM	LoD: 5 μM	95
					S: 0.7 μA μM^−1^ cm^−2^	
	CPE/CeO_2_ NPs	Dopamine	Green synthesis	—	LoD: 0.5 mM	93
	GCE/MWCNTs/CeO_2_-PEDOT	Dopamine	—	FESEM, TEM, XRD, FTIR	LoD: 0.03 μmol L^−1^	96
	GCE/(MC–CeNPs)	Hydroquinon, catechol	—	SEM-EDX, TEM, XPS	—	98
	CeO_2_(CeO_2_/NB) nanohybrid	Hydrazine	Ultrasonic irradiation of Ce(NO_3_)_2_	UV-vis and FTIR, XRD, SEM, HRTEM, Brunauer–Emmett–Teller	LoD: 3.79 × 10^- 9^ M	103
	CuO–CeO_2_ MOFs	Malathion	Direct pyrolysis	SEM, XRD, EDS	LoD: 3.0 nmol L^−1^	104
	Cu CeO_2_ NP/GCE	Purine and pyrimidine	Microwave irradiation	XRD, Raman	—	115
	GCE/CeO_2_–CuO	Nitrite	Precipitation	XRD, UV-Vis, TG-DTA, PL, HRTEM, XPS, FTIR, CV	LoD: 3.3 fM	105
CPE	CPE/Pt/CeO_2_@Cu_2_O	Dopamine	—	PXRD, TEM, SEM	—	106
GCPE	Sn–CeO_2_Np/GCPE	Dacarbazine	Sol–gel	XRD	LoD: 0.01 mM	107
					S: 356.3 μA cm^−2^ mM^−1^	
SPE	CeNPs/SPE	Total antioxidant capacity	—	UV-Vis, electrochemistry	LoD: 0.019 μM	110
					S: 2169.8 μA μM^−1^ cm^−2^	
	SPE–CeNP	Oxymetazoline hydrochloride	—	SEM TEM	LoD: 3 ppm	111
SPCE	Poly(acrylic acid)-coated nanoceria	Fe(CN)_6_^4−^	—	HRTEM	LoD: 57 nM	99
					S: 484.86 μA mM^−1^ cm^−2^	
AuNP-ITO	PNC (poly acrylic acid-coated nanoceria)	Norepinephirine	Precipitation	FTIR	—	116

### 4.2 Electrochemical biosensors

Biosensors are analytical devices that utilize biochemical reactions to detect target analytes. Biosensors contains biochemical compounds that recognize the targeted analytes. In order to improve the performance of biosensors, various nanomaterials, *e.g.*, carbon nanotubes, graphene nanosheets, metal nanoparticles, metal oxide nanoparticles, and their nanoconjugates were incorporated within the biosensors.^117–121^ Metal nanoparticles and metal oxides have been widely used in biosensors to amplify signals and increase surface area, as well as employed as stabilizers for biological receptors.^122^ Electrochemical behavior of nanoceria towards the oxidation and reduction of H_2_O_2_ results in a highly sensitive H_2_O_2_ detection. Table 2 shows the applications employing nanoceria in biosensing in the last four years (2017–2020).

**Table 2:** Nanoceria-based electrochemical biosensors introduced in the last four years (2017–2020)

Method-based on	Electrode modification	Sensing element	Target	Ref.
Enzymatic	Pt/ZnO–CeO_2_/AChE/chitosan	Acetylcholinesterase	Thiocholine	123
	SPCE/AChE/OMC-CS/CeO_2_-CS	Acetylcholinesterase	Pesticide	124
	GCE/CeO_2_NPs-GO	Phytase	Phytic acid	125
	SCPE/(CeO_2_)/(BMIMNO_3_)/tyrosinase	Tyrosinase	Phenolic compounds	126
	CNT (IJPCNT)/(ACeO_2_@GNR/IJPCNT)	Amidase	Acetaminophen	127
	GE/CeO_2_/chitosan/lactate oxidase	Lactate oxidase	Lactic acid	129
	GE/Au@CeMOF-cDNA	Telomerase	Hidroquinone	128
	FTO/CeO_2_/GO_*x*_	Glucose oxidase	Glucose	130
Antibody	CeO_2_/FeO_*x*_@mC_500-900_	Anti CA19-9	Carbohydrate antigen 19-9	135
	GCE/AuNPs/Ab_1_, and Co_3_O_4_@CeO_2_Au@Pt/Ab_2_	Anti SCCA	Squamous cell carcinoma antigen	140
	Cu_2_O@CeO_2_–Au/Ab	Anti PSA	Prostate specific antigen	131
	SPE/GO–CeO_2_–CS	Anti AFM_1_	Aflatoxin M_1_	132
	AuNPs/Ab_1_, and CeO_2_–MoS_2_/Pb^2+^/Ab_2_	Anti CEA	Carcinoembryonic antigen	133
	CeO_2_–CdS, and SiO_2_/PDA-Ag	Anti BNP	Brain natriuretic peptide	134
	SPCE-Au/CeO_2_-anti HER2	Anti HER2	HER2	30
DNA	CeO_2_ NR/DNA	DNA	*Salmonella*	136
	GCE/CeO_2_ NR/chitosan/DNA	DNA	*Clostridium perfringens*	137
	CeO_2_/DNA amplicons-targets	PCR amplicons	Glucose	138
	MPBA-SiO_2_@Au/dsDNA/CeO_2_	dsDNA	Glycoprotein ovalbumin	139
	GCE/AuNPs@Fe-MOFs/SA/bio-CP/BSA	DNA	CYP2C19*2 allele	141

#### 4.2.1 Enzyme-based electrochemical biosensor

A Pt/ZnO–CeO_2_/AChE/chitosan based biosensor was developed for sensitive quantification of thiocholine with acetylcholinesterase (AChE) enzyme. AChE interacts with substrate of acetylthiocholine chloride (ATCl) and produces an electroactive product that leads a clear oxidation peak. In order to prepare ZnO–CeO_2_/AChE/chitosan nanocomposite, different percentages of ZnO, CeO_2_, and chitosan were dispersed in AChE coated onto Pt electrode's surface. The biosensor showed high electron transfer rate, biocompatibility, and good conductivity.^123^ An AChE enzyme biosensor was developed for the detection of organophosphorus pesticide residue using cerium oxide-chitosan (CeO_2_–CS) modified SPCEs and mesoporous carbon-chitosan (OMC–CS). CeO_2_–CS was dropped on the SPCE surface, and CeO_2_–CS/SPCE was modified with OMC–CS. Finally, AChE was immobilized onto SPCE to obtain AChE/OMC–CS/CeO_2_–CS/SPCE. The proposed AChE biosensor exhibited a good reproducibility and high stability.^124^ The phytase enzyme immobilization on a chemically modified cerium oxide nanoparticles decorated graphene oxide (CeO_2_NPs–GO) on the surface of GCE was used for the detection of phytic acid (PA). The decrease in the current for the ferricyanide cyanide solution with PA concentration on the CeO_2_NPsGO electrode surface was elucidated in the range between 0.066 and 6.6 ppb. The developed PA biosensor provided a much lower detection limit (LOD = 0.001 ppb) compared to previously reported electrochemical techniques for PA detection.^125^

**Fig. 13:**
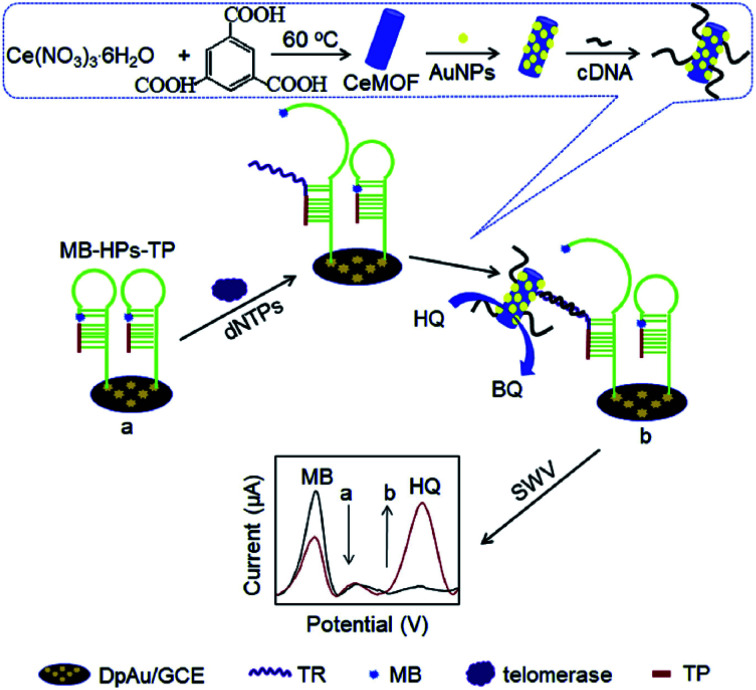
Experimental steps of the detection of telomerase activity based on catalysis of Au@CeMOF toward HQ oxidation and conformation switch of MB-labeled hairpin DNA. Reprinted from ref. 123 with permission © 2020 Elsevier B.V.

Tyrosinase modified cerium oxide (CeO_2_)/1-butyl-3-methylimidazolium nitrate composite was deposited on SPCE for the detection of phenolic compounds. In this technology, BMIMNO_3_ was used to enhance electron transfer of medium to the electrode surface and the conductivity of the electrodes. The developed biosensor possesses an LOD and sensitivity, *e.g.*, 0.1221 μM and 80.86 nA μM^−1^, respectively.^126^ An amidase/cerium dioxide@graphene nanoribbon composite modified inkjet-printed carbon nanotube electrode (ACeO_2_@GNR/IJPCNT) was constructed for the detection of acetaminophen. IJPCNT electrodes had small sample volume, *e.g.*, 5 μL, corporates amidase A enzyme that showed a high selectivity and sensitivity. The proposed biosensor resulted in a broad linear range of concentration between 1 and 100 μM, and a low limit of detection of 0.18 μM of acetaminophen.^127^ A ratiometric electrochemical biosensor based on gold nanoparticles functionalized with cerium-based metal–organic frameworks tagged cDNA (Au@CeMOF-cDNA) have been developed for telomerase activity. CeMOFs was formed by a green method synthesized of cerium nodes), where 1,3,5-benzenetricarboxylic acid was used as a linker. Initially, methylene blue (MB) modified hairpin DNA probe and telomerase primer (TP) immobilized on the glassy carbon-deposited gold electrodes (GCE-DpAu) *via* Au–S attachment. In the presence of telomerase and dNPs, the extended TP could open DNA hairpin, and keep the MB away from the electrode surface, which results in a decrease in the electrochemical signal. Meanwhile Au@CeMOF-cDNA was captured by DNA probe *via* hybridization, and leading to an increase in electrochemical signal due to its electrocatalytic behavior toward hydroquinone (HQ) oxidation. The experimental steps is shown in Fig. 13. The proposed biosensor showed a wide range of telomerase activity between 2 × 10^2^ and 2 × 10^6^ cells per mL with a detection limit of 27 cells per mL.^128^

A higher catalytic of nanoceria embedded in amorphous carbon and associated with chitosan–lactate oxidase was offered to employ it as a lactic acid biosensor. Oxidation of lactic acid by lactate oxidase produced by H_2_O_2_ was measured with CV on the gold electrode. The sensor showed a wide linear range from 3 to 300 μM with a low detection limit of 300 nM lactic acid.^129^ Cerium oxide based on GO_*x*_ enzyme that immobilizes through electrostatic interaction on the fluorine doped tin oxide (FTO) electrode modified CeO_2_ nanostructures. The developed biosensors showed a sensitivity between 208 and 2290 μA cm^−2^ mM^−1^, with a detection limit of 1 nM. Increased surface area and high defect concentration on the surface enhance performance of the proposed biosensor.^130^

#### 4.2.2 Electrochemical immunosensors

Antibody-based electrochemical biosensors, in other words, immunosensors use antibodies as capturing agents. Immunosensors possess high sensitivity thanks to numerous nanomaterials that facilitate electron transfer rate and carrying capacity.^129^ For example, bimetallic cerium and ferric oxide nanoparticles embedded in mesoporous carbon matrix (CeO_2_/FeO_*x*_@mC) was employed for the detection of carbohydrate antigen 19-9 (CA19-9) with EIS. Calcination at different temperatures within highly graphitized mesoporous carbon matrix was performed to obtain bimetallic Ce–Fe-based metal organic framework (CeFe-MOF). CA 19-9 antibody was anchored to CeO_2_/FeO_*x*_@mC network *via* ester-like bridging between carboxylic groups of antibody. LOD was found as 10 μU mL^−1^ within a broad range, *e.g.*, from 0.1 mU mL^−1^ to 10 U mL^−1^.^129^ A core–shell nanocomposite amino functionalized cuprous oxide@ceric dioxide (Cu_2_O@CeO_2_–NH_2_) was introduced for the detection of prostate specific antigen (PSA). Such substrate possess btter electrocatalytic activity *via* the reduction of H_2_O_2_ and the developed Cu_2_O@CeO_2_–Au based immunosensor provided a low detection limit, *e.g.*, 0.03 pg mL^−1^ PSA.^131^

SPEs modified with graphene oxide–chitosan (GO–CS) and cerium oxide–chitosan (CeO_2_–CS) were designed for the detection of aflatoxin M1 (AFM1). The synergic effects of GO, nanoceria, and chitosan resulted in strong electrical conductivity, good redox property, and strong complexation. Furthermore, it accelerated electron transfer, and allowed larger numbers of anti-AFM1 antibody (ab-AFM1) immobilization. DPV and CV characterization tests showed a detection limit, *e.g.*, 0.009 μg L^−1^ AFM1.^132^ A nanohybrid of molybdenum disulphide with cerium oxide (CeO_2_–MoS_2_) was developed to absorb lead ions (Pb^2+^), attaching to an capturing antibody (Ab_2_) for the detection of carcinoembryonic antigen (CEA). Square wave voltammetry measurements employing the Pb^2+^ electrical signal demonstrated a limit of detection, *e.g.*, 0.3 pg mL^−1^ of CEA.^133^ A sandwich type photoelectrochemical (PEC) immunosensor was developed for the detection of brain natriuretic peptide (BNP). The photoactive CeO_2_–CdS platform employs SiO_2_/PDA-Ag nanocomposites for signal amplification. Using CeO_2_–CdS further enhanced photocurrent responses thanks to the well matched energy band. In order to improve the sensitivity of the immunosensor, Ag nanoparticles (AgNPs) were used due to their ability to anchor antibodies *via* the chemical bonding between AgNPs and –NH_2_ of antibodies. This PEC immunosensor exhibited a low detection limit, *e.g.*, 0.05 pg mL^−1^ within a range between 0.1 pg mL^−1^ and 5 ng mL^−1^. The immunosensor exhibited good stability, reproducibility and specificity, which could be applicable for the detection of different biomarkers.^134^ AuNP decorated CeO_2_–CuO corporated with Au@Ag-thionine (signal label) was constructed for the detection of procalcitonin (PCT). The proposed sandwich immunosensor provided a detection limit of 0.17 pg mL^−1^ PCT within a linear range between 0.5 pg mL^−1^ and 50 ng mL^−1^ with.^135^

#### 4.2.3 DNA-based electrochemical biosensor

A CeO_2_ NR was developed to detect *Salmonella* pathogen. Impedimetric measurements exhibited a linear range of target DNA concentration, *e.g.*, between 0.01 μM and 2 μM, with a limit of detection and sensitivity, 0.01 μM and 3362.1 Ω μM^−1^ cm^−^^2^, respectively.^136^ CeO_2_ NR also exhibited a strong adsorption to DNA, and was employed as an electrochemical biosensor for the detection of *Clostridium perfringens.* The DNA probe immobilized on the CeO_2_ NR/chitosan modified GCE *via* metal coordination bonding. Impedimetric tests showed a limit of detection 7.06 × 10^−15^ mol L^−1^ of target DNA in a linear range between 10^−14^ and 10^−7^ mol L^−1^.^137^ Electrostatic interaction between nanoceria and DNA was employed in a personal glucose meter (PGM) based on glucose oxidase-like activity that enabled correlation of DNA amplicon with glucose level. The biosensor is based on inducing aggregation of DNA amplicon, target DNA, and nanoceria that reduces the efficiency of nanoceria-catalyzed glucose oxidation reaction. PGM measured the level of nanoceria-suppressed conversion of glucose to gluconic acid. The proposed biosensor provided high selectivity and sensitivity.^138^ 4-mercaptophenylboronic acid (MPBA) molecularly imprinted polymer that is modified with CeO_2_ and SiO_2_@Au nanocomposites were developed as a signal tag in DNA-based electrochemical biosensor for the detection of glycoprotein ovalbumin (OVA). A nicked double-strand DNA was added to form SiO_2_@Au/dsDNA/CeO_2_, and immobilized on the electrode surfaces. Using the developed biosensor, a broad linear range, *e.g.*, between 1 pg mL^−1^ and 1000 ng mL^−1^, and a low detection limit, 0.87 pg mL^−1^ OVA were obtained.^139^

## 5 Conclusion

Nanoceria can be synthesized with various physical and chemical methods including precipitation, hydrothermal, and combustion, and bio- or green synthesis methods. Nanoceria morphologies including size, shape, and distribution can spectroscopically characterized, *e.g.*, UV-VIS, FTIR, Raman spectroscopy, photoluminescence, microscopically characterized, *e.g.*, SEM, FESEM, TEM, HERTEM, X-ray techniques, *e.g.*, XRD, XPS, and thermal stability tests, *e.g.*, TGA, DTA/DTG. Among the metal oxide-based nanoparticles, nanoceria or cerium oxide nanoparticles attracted significant attention that can be used as an electrochemical sensor thanks to their high mechanical strength, oxygen ion conductivity, oxygen storage capacity, high chemical stability, and non-toxicity. Their structure exhibit strong oxygen storage capacity due to the high numbers of oxygen vacancy defects in their crystal structure. Nanoceria can also act as oxidizing and reducing agents due to the oxidation state of cerium oxide nanoparticles that can vary between +3 and +4. Nanoceria is widely used in biosensing, *e.g.*, acting as catalysts to mimic activity of enzymes in biosensors. Furthermore, the combination of nanoceria with different nanomaterials was realized to improve sensor performance. Vast numbers of nanomaterials were employed in electrochemical sensing with various electrodes and analytes. Biocompatibility of nanoceria helps immobilizing biomolecules with low iso-electric points through electrostatic interactions. Nanoceria also enhances sensitivity derived from various signal strengthening strategies.

## Conflicts of interest

The authors declare that there is no conflict of interests regarding the publication of this paper.

## Supplementary Material
