# Correction: Synthesis and characterization of nanoceria for electrochemical sensing applications

**DOI:** 10.1039/d1ra90121d

**Published:** 2021-05-24

**Authors:** Yeni Wahyuni Hartati, Seda Nur Topkaya, Shabarni Gaffar, Husein H. Bahti, Arif E. Cetin

**Affiliations:** Department of Chemistry, Faculty of Mathematics and Natural Sciences, Universitas Padjadjaran Indonesia yeni.w.hartati@unpad.ac.id; Department of Analytical Chemistry, Faculty of Pharmacy, Izmir Katip Celebi University Turkey; Izmir Biomedicine and Genome Center Izmir Turkey

## Abstract

Correction for ‘Synthesis and characterization of nanoceria for electrochemical sensing applications’ by Yeni Wahyuni Hartati *et al.*, *RSC Adv.*, 2021, **11**, 16216–16235, DOI: 10.1039/D1RA00637A.

The authors regret that the figures throughout the article were incorrectly referenced. The correct references for each figure are described in Table 1:

**Table 1:** Corrected references

Figure	Original reference	Corrected reference
Fig. 1	47	53
Fig. 2	51	(Missing)
		H. Jan, M. A. Khan, H. Usman, M. Shah, R. Ansir, S. Faisal, N. Ullah and L. Rahman, *RSC Adv.*, 2020, **10**, 19219–19231
Fig. 3	75	80
Fig. 4	76	81
Fig. 5	77	82
Fig. 6	42	48
Fig. 7	80	85
Fig. 8	29	29
Fig. 9	81	86
Fig. 10	88	93
Fig. 11	93	98
Fig. 12	107	112
Fig. 13	123	128

The Royal Society of Chemistry apologises for these errors and any consequent inconvenience to authors and readers.

## Supplementary Material

